# Optimizing urine albumin-to-creatinine ratio testing and referral pathways for chronic kidney disease: a nominal group technique consensus study among Italian experts

**DOI:** 10.1007/s40620-025-02371-w

**Published:** 2025-08-20

**Authors:** Irene Capelli, Michele De Benedictis, Andrea Di Lenarda, Vittorio Di Maso, Paolo Fabbrini, Paola Galli, Carlo Garofalo, Antonio Maria Leone, Maria Ida Maiorino, Marita Marengo, Sara Pasqualetti, Francesco Pesce, Alberto Polimeni, Michele Provenzano, Danilo Ribichini

**Affiliations:** 1 Department of Medical and Surgical Sciences (DIMEC), Alma Mater Studiorum, University of Bologna, Bologna, Italy; 2 Nephrology, Dialysis and Kidney Transplant Unit, IRCCS Azienda Ospedaliero-Universitaria Di Bologna, Bologna, Italy; 3 S.C. Cardiologia, Dipartimento Medico Specialistico, ASLCN1 Savigliano, Cuneo Italy; 4 Cardiovascular Center, University Hospital and Health Services of Trieste, Trieste, Italy; 5 Nephrology and Dialysis Unit of Trieste, University Hospital and Health Services of Trieste (ASUGI), Trieste, Italy; 6 Nephrology and Dialysis Unit, ASST Nord Milano, Ospedale Bassini, Cinisello Balsamo, Milan Italy; 7 Diabetologia E Malattie Metaboliche ‘Giovanni Segalini’, ASST Nord Milano, Ospedale Bassini, Cinisello Balsamo, Milan Italy; 8 Division of Nephrology, University of Campania “Luigi Vanvitelli”, Naples, Italy; 9 Centro Di Eccellenza in Scienze Cardiovascolari, Ospedale Isola Tiberina, Gemelli Isola, Università Cattolica del Sacro Cuore, Rome, Italy; 10 Department of Advanced Medical and Surgical Sciences, Unit of Endocrinology and Metabolic Diseases, University of Campania “Luigi Vanvitelli”, Naples, Italy; 11 S.C. Nefrologia e Dialisi, Dipartimento Medico Specialistico, ASLCN1 Cuneo, Italy; 12 Clinical Pathology Unit, Luigi Sacco University Hospital, ASST Fatebenefratelli-Sacco, Milan, Italy; 13 Department of Translational Medicine and Surgery, Università Cattolica del Sacro Cuore, Rome, Italy; 14 Division of Renal Medicine, Ospedale Isola Tiberina-Gemelli Isola, Rome, Italy; 15 Department of Pharmacy, Health and Nutritional Sciences, University of Calabria, Rende, Italy; 16 Division of Interventional Cardiology, Annunziata Hospital, Cosenza, Italy; 17 Nephrology, Dialysis and Renal Transplant Unit, Department of Pharmacy, Health and Nutritional Sciences, University of Calabria, Rende, Hospital ‘SS. Annunziata’, Cosenza, Italy; 18 Division of Endocrinology and Diabetes Prevention and Care, IRCCS Azienda Ospedaliero-Universitaria Di Bologna, Bologna, Italy

**Keywords:** Chronic kidney disease, uACR evaluation, Cardiovascular risk, Nephrology referral

## Abstract

**Background:**

Chronic kidney disease (CKD) represents a major global health burden, often diagnosed at advanced stages when treatment is less effective. Albuminuria, assessed by the urine albumin-to-creatinine ratio (uACR), is a key biomarker for CKD detection and risk stratification. Despite guideline recommendations, adherence to uACR testing remains low, limiting early diagnosis and timely referral. The ALLIANCE project aimed to develop a multidisciplinary consensus on optimizing uACR testing and referral pathways for improved CKD management in at-risk populations.

**Methods:**

A modified nominal group technique was used to achieve expert consensus. Seven nephrologists and eight specialists in other disciplines  (cardiologists, endocrinologists, diabetologists, and a clinical biochemist) participated in structured discussions and ranked statements across three domains: (1) at-risk population definition, (2) barriers to uACR testing, and (3) CKD management and referral. Relevance rankings were analyzed using hierarchical clustering.

**Results:**

Twenty-seven consensus statements were developed, eight of which were deemed highly relevant. Key recommendations included expanding CKD risk awareness to encompass obesity and family history, enhancing clinician education, and improving coordination between nephrologists and other specialists. Early nephrology referral was emphasized for patients with marked albuminuria, rapid renal decline, or specific risk factors. Integration of digital health tools, including shared electronic health records, was advised to support coordinated care.

**Conclusions:**

The ALLIANCE project identified critical gaps in CKD detection and management. Addressing these through clinician education, standardized uACR testing protocols, and multidisciplinary collaboration may improve outcomes and reduce cardiorenal risk. Implementation of these consensus recommendations could facilitate earlier diagnosis and better management of high-risk patients.

**Graphical abstract:**

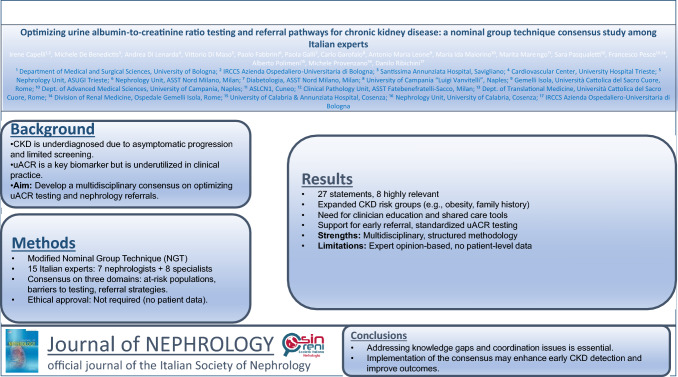

## Introduction

The Global Burden of Disease study ranked chronic kidney disease (CKD) as the 10th leading cause of death in 2019, with projections suggesting it will become the 5th cause by 2040 [1]. Despite this, most CKD diagnoses occur in advanced stages, where intervention is less effective [2]. Early disease detection is often hindered by the asymptomatic nature of CKD and insufficient routine screening [3]. Moreover, a clear etiological diagnosis of CKD is often lacking.

CKD is defined in the Kidney Disease: Improving Global Outcomes (KDIGO) guidelines as the presence of kidney structural or functional abnormalities for at least 3 months, with potential health implications [4]. It is classified based on the cause, glomerular filtration rate (GFR; G1–G5), and albuminuria category (A1–A3), collectively known as the CGA classification [4]. The criteria for CKD diagnosis include an estimated GFR (eGFR) < 60 mL/min/1.73 m^2^ for over 3 months or a urine albumin-to-creatinine ratio (uACR) > 30 mg/g. Thus, both albuminuria and reduced eGFR serve as key biomarkers for CKD detection and monitoring. Furthermore, they are recognized as independent predictors of cardiovascular risk. Notably, an elevated uACR is a significant cardiorenal risk factor, even when eGFR remains within normal ranges [5, 6].

Based on this evidence, different guidelines recommend uACR screening for high-risk groups, such as patients with diabetes, hypertension, and cardiovascular disease (CVD) [4, 7]. However, real-world data reveal suboptimal screening practices, with uACR screening performed in a minority of patients with type 2 diabetes and in a small proportion of hypertensive patients [8–10]. In addition, recent European data show substantial variability in CKD referral practices, with marked differences in the proportion of patients referred to nephrologists across 27 countries, underscoring the need for clearer referral criteria and improved awareness among non-nephrologist clinicians [9].

The ALLIANCE (ALbuminuria and cardio-renal risk: a new roAdmap for ideNtification and optimization of CKD patients) project was designed to address these gaps. In particular, the project aimed to develop a multidisciplinary consensus document for optimizing uACR testing, identifying barriers and knowledge gaps, and defining the proper timing for nephrology referrals.

## Methods

### Project overview

The ALLIANCE project was designed to develop a multispecialty document to improve uACR testing and CKD management in at-risk patients. An expert panel composed of seven nephrologists, alongside eight specialists (cardiologists, endocrinologists, diabetologists, and a clinical biochemist), participated in the project.

A modified version of the nominal group technique (see the next paragraph for details) was utilized to gather expert opinions and collect statements on three main areas of interest, which were preliminarily defined by the seven nephrologists during an online meeting held in June 2024. The three identified areas were: (1) definition of at-risk populations for CKD, (2) barriers to uACR testing, and (3) management and referral of CKD patients.

**Fig. 1:**
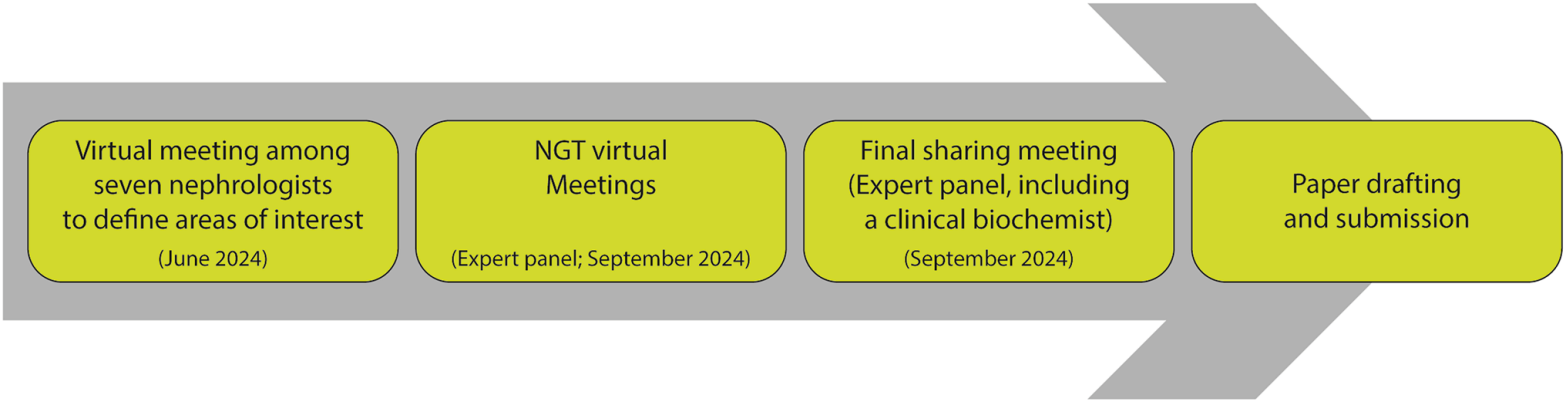
Project workflow

The final nominal group technique results were shared and discussed among the experts during an online meeting in September 2024 and were used to draft the present paper. In addition, the expert opinion of a clinical biochemist was sought to provide a critical evaluation of the nominal group technique results. All meetings, discussions, and surveys were conducted in Italian. The study was not prospectively registered. Figure 1 summarizes the project workflow.

### Nominal group technique

The nominal group technique is an expert-based, structured, and direct method used to facilitate meetings aimed at making decisions on specific topics lacking strong evidence. It is commonly applied in consensus studies. The classical nominal group technique process comprises four phases: (1) silent generation, (2) thought sharing, (3) statement definition, and (4) statement ranking. A modified nominal group technique was used within this project.

In detail, the experts were asked to develop their own statements based on the three predefined areas of interest. Experts were selected based on their documented clinical expertise in chronic kidney disease, with particular reference to albuminuria assessment and nephrology referral. Selection criteria included prior involvement in CKD-related clinical initiatives and/or authorship of peer-reviewed publications on relevant topics. Seven online meetings (1 h each) were conducted, each including a nephrologist, one of the specialists, and a methodologist. During these sessions, the experts provided their opinions, which were then discussed, refined, and converted into statements by the methodologist. After the seven nominal group technique meetings, the experts had the opportunity to review and refine their statements. The final list of statements was included in an online survey for ranking. The experts ranked the statements based on their relevance using a five-point Likert scale (1 = not relevant to 5 = extremely relevant). The voting was conducted via the online survey platform SurveyMonkey in September 2024. For each statement, the individual relevance scores assigned by participants were summed to obtain a cumulative score. The scores were then subjected to a hierarchical clustering process, which grouped the statements into three different tiers according to relevance: tier 1: high relevance; tier 2: intermediate relevance; and tier 3: low relevance. Statements with the highest cumulative relevance scores were selected as key messages for inclusion in this document.

### Statistical analysis

Descriptive statistics were applied to the data collected during the ranking phase of the nominal group technique process. The sum of scores for each statement was used to determine its level of relevance (low, intermediate, or high). No inferential statistical analysis was carried out.

## Results

The nominal group technique process produced 27 statements across the three thematic areas (seven in area 1, eight in area 2, and 12 in area 3). Overall, eight statements received high relevance scores (three in area 1, two in area 2, and three in area 3). A total of 11 statements received intermediate relevance scores, with sums only marginally lower than those of the highly relevant statements, suggesting comparable clinical significance. The results of the nominal group technique process are reported in Tables 1, 2 and 3 and described in the paragraphs below.

**Table 1:** Nominal group technique results on the definition of at-risk populations for chronic kidney disease

Statement Number	Statement	Sum of scores	Relevance
1.1	Patients must be aware of CKD risk factors, such as hypertension, smoking, and diet, to effectively reduce them	61	High
1.2	The definition of at-risk patients for CKD is clear, and the current KDIGO 2024 guidelines provide a comprehensive framework. However, risk awareness across medical specialties remains limited, particularly with the introduction of new therapies	58	High
1.3	In addition to the “traditional” groups at risk of CKD (patients with diabetes, hypertension, or cardiovascular disease), other categories should be considered, including those with a family history of kidney disease (including genetic predisposition) and patients with obesity	55	High
1.4	CKD risk varies across different patient populations, and eGFR–uACR correlation tables should be customized based on patient characteristics, treatments, and interventions	52	Intermediate
1.5	The connection between cardiovascular and renal health persists even after acute kidney injury has resolved, increasing cardiovascular risk	52	Intermediate
1.6	The number of patients at risk of CKD from iatrogenic causes is increasing (e.g., patients exposed to nephrotoxic oncology drugs or complex antibiotic therapy), as are patients at risk of CKD from occupational causes (e.g., exposure to metals)	52	Intermediate
1.7	Diabetic patients can experience a prolonged “silent” phase (8–10 years without symptoms), during which early complications, such as retinopathy, may indicate a higher risk of CKD	48	Low

**Table 2:** Nominal group technique results on barriers to using urine albumin-creatinine ratio (uACR) testing

Statement Number	Statement	Sum of scores	Relevance
2.1	The main barriers to widespread uACR testing are cultural and educational rather than logistical. Clinicians need greater awareness of the importance of measuring both albuminuria and creatinine to obtain uACR. Standardization of testing methods should be promoted to facilitate broader adoptions of uACR testing	62	High
2.2	GPs should routinely request uACR testing for at-risk patients before referring them to specialists	62	High
2.3	A uACR ≥ 300 mg/g indicates a high risk of CKD progression and must be universally recognized and addressed in clinical practice	60	Intermediate
2.4	uACR should be directly requested in clinical practice to prevent errors rather than prescribing albuminuria and creatinine separately	58	Intermediate
2.5	Obsolete terms such as “microalbuminuria” and “macroalbuminuria” should be replaced with the more precise and standardized uACR	58	Intermediate
2.6	uACR values should always be reported in mg/g (mg/mmol) for consistency, with abnormal values clearly indicated in laboratory reports	56	Low
2.7	Laboratories should standardize uACR testing using first-morning urine samples and calculate the ratio based on multiple samples when necessary	54	Low
2.8	Patients should be instructed on proper uACR testing methods (e.g., collecting a first-morning urine sample, avoiding testing during menstruation or after intense physical activity)	52	Low

**Table 3:** Nominal group technique results on management and referral of CKD patients

Statement Number	Statement	Sum of Scores	Relevance
3.1	The implementation of automated coordination tools, including telemedicine, would improve communication among nephrologists and other specialists and facilitate the shared management of treatments	60	High
3.2	Early CKD management significantly reduces hospitalizations and enhances the effectiveness of nephroprotective therapies	59	High
3.3	The adoption of shared electronic health records would facilitate coordinated follow-up and improve the efficiency of patient management	59	High
3.4	Complications such as metabolic acidosis and hyperparathyroidism require nephrologist-prescribed medications, making a nephrology referral necessary	57	Intermediate
3.5	A multidisciplinary approach, including regular specialist meetings, is beneficial for optimizing patient care	57	Intermediate
3.6	CKD progression must be closely monitored: a decline in GFR greater than 3–4 mL/min/year requires a nephrology referral	56	Intermediate
3.7	Patients with eGFR < 45 mL/min/1.73 m^2^ or albuminuria (A2 level) should be referred to a nephrologist	55	Intermediate
3.8	Patients meeting specific criteria – such as polycystic kidney disease, eGFR < 60 mL/min/1.73 m^2^ in association with micro- or macroalbuminuria (uACR > 30 mg/g), or active urinary sediment – should be referred to a nephrologist	55	Intermediate
3.9	Patients receiving treatments that may affect CKD progression and who have impaired renal function must be followed by a nephrologist	51	Low
3.10	Each patient should have a single specialist as a primary point of contact, coordinating with other specialists as needed to avoid confusion	50	Low
3.11	Patients in stage G3a/A1 with increased cardiovascular risk but low risk of renal progression can be managed by GPs	49	Low
3.12	Patients with moderate CKD risk do not necessarily require a nephrology consultation	45	Low

### Definition of at-risk populations for chronic kidney disease

Highly relevant statements (1.1, 1.2, and 1.3; Table 1) emphasized the need for improved CKD risk awareness among both patients and healthcare professionals. They also highlighted the importance of considering additional “at-risk” groups, such as patients with a family history of kidney disease – including genetic predisposition – and those with obesity.

Three statements were categorized as having intermediate relevance (1.4, 1.5, and 1.6; Table 1). These addressed variability in CKD risk across different patient populations, suggesting that eGFR–uACR correlation tables should be tailored based on patient characteristics, treatments, and interventions. The experts also highlighted the cardiovascular–renal connection, stressing that cardiovascular risk remains elevated even after acute kidney injury has resolved. Furthermore, the increasing number of patients at risk of CKD due to iatrogenic causes (e.g., exposure to nephrotoxic oncology drugs) and occupational exposures (e.g., metals) was recognized as an important emerging issue.

### Barriers to using the uACR test

Highly relevant statements (2.1 and 2.2; Table 2) identified cultural factors as the primary barrier to uACR testing, emphasizing the need for enhanced clinician education. Additionally, they suggested that general practitioners (GPs) should routinely request uACR testing for at-risk patients before referring them to specialists. Statements of intermediate relevance (2.3, 2.4, and 2.5; Table 2) proposed simplifying testing protocols to prevent errors. They also emphasized that a uACR ≥ 300 mg/g indicates a high risk of CKD progression, which becomes very high when eGFR is < 45 mL/min/1.73 m^2^ and must be universally recognized and addressed in clinical practice. Furthermore, the experts advocated eliminating obsolete terminology such as “microalbuminuria” and “macroalbuminuria” in favor of graded uACR, a more precise and standardized measure.

### Management and referral of CKD patients

Highly relevant statements (3.1, 3.2, and 3.3; Table 3) emphasized the importance of implementing automated coordination tools (e.g., telemedicine) to enhance communication between nephrologists and other specialists, facilitating shared medication management. Additionally, early CKD management was highlighted as critical for reducing hospitalizations and improving the effectiveness of nephroprotective treatments. The use of shared electronic health records to ensure coordinated follow-up and improve patient care efficiency was also deemed highly relevant.

Statements of intermediate relevance (3.4, 3.5, 3.6, 3.7, and 3.8; Table 3) highlighted the necessity of nephrology referral for patients with specific risk factors, including polycystic kidney disease, eGFR < 60 mL/min/1.73 m^2^ in association with uACR > 30 mg/g, or active urinary sediment, and the management of complications such as metabolic acidosis, hyperparathyroidism, and anemia, which require nephrologist-prescribed medications. Nephrology care was considered essential regardless of the underlying cause of CKD. The experts also supported a multidisciplinary approach to CKD management, with regular meetings between specialists to optimize patient care. Monitoring CKD progression and referring patients when GFR declines by more than 3–4 mL/min/year was identified as a key strategy for effective disease management.

## Discussion

The ALLIANCE project aimed to develop a multispecialty consensus document to address gaps in CKD screening and management, focusing on three key areas: defining at-risk populations, improving the use of uACR testing, and optimizing nephrology referral pathways. The goal was to create clear, actionable recommendations to enhance early detection and management of CKD, particularly in high-risk patients.

As emphasized in previous studies, albuminuria is a well-established marker not only for CKD progression but also for CVD risk, yet it remains underutilized in clinical practice [6]. An accurate initial diagnosis of CKD is fundamental to optimizing the patient’s therapeutic pathway. In this context, albuminuria serves not only as a prognostic biomarker but also as a key diagnostic marker, essential for risk stratification and guiding clinical management. According to the 2024 KDIGO guidelines, the initial assessment of CKD should include uACR measurement, as elevated albuminuria identifies patients at risk for disease progression and cardiovascular events at an early stage [4]. KDIGO recommends that a single elevated uACR value should not be considered diagnostic, and confirmation with a repeat test—preferably using a first-morning void urine sample—is advised to reduce variability and the risk of false-positive results [4]. However, integrating albuminuria into diagnostic algorithms enables more precise risk stratification and supports early and effective therapeutic decisions.

Traditionally, the gold standard for detecting and quantifying albuminuria has been 24 h urine collection; however, this method is cumbersome and subject to over- and under-collection errors. Instead, a spot urine uACR measurement correlates well with the 24 h albumin excretion. uACR is measured from a first-morning void urine sample and calculated as milligrams of albumin per gram of urine creatinine (mg/g). This calculation is based on the mechanistic assumption that urinary creatinine excretion rate approximates 1 g/day in patients with eGFR < 60 mL/min/1.73 m^2^, meaning that uACR reflects the 24 h albumin excretion.

Results from the nominal group technique process underscored the urgent need for systematic uACR testing in high-risk populations, including those with diabetes, hypertension, and CVD, consistent with the 2024 KDIGO recommendations [4]. Moreover, additional “at-risk” patient categories were suggested, namely, individuals with a family history of kidney disease (including genetic predisposition) and those with obesity. In particular, obesity must be recognized as a multisystem disease affecting the kidneys, reinforcing the need for early obesity management to prevent CKD progression.

Despite the availability of international guidelines recommending routine albuminuria testing, underuse of this test continues to hinder timely CKD detection and management [10]. Nominal group technique data identified significant barriers to the wide adoption of uACR testing, particularly educational and cultural factors among clinicians. In line with literature evidence, many clinicians reported not prioritizing uACR testing in routine assessments of high-risk patients due to a lack of awareness regarding its diagnostic value and perceived irrelevance to patient management decisions [10]. To bridge this gap, clinical laboratories can play a proactive role in improving uACR utilization. For instance, they could develop local guidelines for proper urine sample collection, emphasizing that first-morning void urine specimens should be preferred and used as confirmatory samples when spot urine results require verification. Awareness of preanalytical factors affecting uACR accuracy is crucial, and laboratory professionals should actively communicate these issues to clinicians [11]. Additionally, uniform national guidelines for uACR test requisition are lacking, and not all clinicians can directly request the test. To overcome this, clinical laboratories should ensure that uACR can be easily requested and reported as a standardized ratio, minimizing technical barriers. In addition, uACR results obtained from the central laboratory using quantitative immunoassays are required to ensure test quality, discouraging the use of semiquantitative or dipstick analysis. Clinical laboratories should also adhere to the recommended unit of measurement for uACR (mg/g) and discontinue obsolete terminology, ensuring that multiple decision thresholds are used to classify uACR values as normal, mildly, or severely increased. Multidisciplinary collaboration between laboratory professionals and clinicians is essential to align uACR testing with clinical needs.

Although timely referral to a nephrologist is crucial for optimal CKD management, particularly when albuminuria is detected, the ALLIANCE project revealed significant variations in referral practices across regions and medical specialties. This inconsistency can lead to delayed referrals, which are associated with poorer patient outcomes due to CKD progression, increased mortality, higher healthcare costs, and more frequent adverse events, particularly kidney failure and, to a lesser extent, major adverse cardiovascular events [9, 12, 13]. Early involvement of nephrology care, combined with consistent uACR testing, can reduce hospitalizations and improve the effectiveness of nephroprotective therapies, particularly in high-risk patients with significant albuminuria or eGFR decline. Recent studies proposed indexing albuminuria or proteinuria to estimated eGFR to improve the prognostic assessment of CKD patients. This approach accounts for the nonlinear relationship between filtration rate and protein excretion, allowing for better discrimination of progression risk. In particular, fractional protein excretion has been shown to predict renal outcomes more accurately in patients with glomerular diseases or preserved eGFR [14–17]. However, educational and cultural gaps among non-nephrologist specialists remain a major barrier to the widespread application of clinical guidelines principles to most high-risk patients. Since early, direct nephrologist involvement is not feasible for all high-risk patients, there is a need to increase awareness among diabetologists, cardiologists, and GPs to enhance uACR testing, establish clinical pathways, and coordinate therapeutic intervention with nephrologists.

A nephrology referral of a patient from primary care should be considered if any of the following conditions are present: (1) eGFR < 45 mL/min/1.73 m^2^; (2) rapid decline in renal function in the last year (≥ 5 mL/min/year); (3) presence of uACR ≥ 200 mg/g; (4) active urinary sediment, defined by the presence of hematuria or red blood cell (RBC) casts (in the absence of urinary tract infection or urological disease); (5) hydroelectrolyte abnormalities (including volume expansion or depletion, abnormal levels of Na^+^, K^+^, Ca^2+^, PO_4_^3−^) or unexplained anemia.

The implementation of multidisciplinary approaches to CKD care, including the integration of digital health tools, was also emphasized. These tools, such as shared electronic health records, were viewed as essential for enhancing communication between nephrologists and other specialists, facilitating a more coordinated approach to CKD management. Multidisciplinary CKD care models have demonstrated significant clinical benefits, including reduced mortality, improved glycemic control, and enhanced patient self-care [18, 19]. A co-designed integrated kidney and diabetes model of care, as reported by Zimbudzi et al., showed that structured collaboration across specialties leads to better disease management and patient engagement, ultimately reducing complications and improving long-term prognosis [20]. These integrated strategies reinforce the need for closer coordination among nephrologists, diabetologists, cardiologists, and primary care providers to optimize CKD management. Furthermore, an integrated and multidisciplinary approach could be particularly advocated in the presence of multimorbidity and frailty, with the aim of shifting from individual specialist-driven management to a shared care model, thus maximizing patient safety (e.g., limiting adverse events) and improving compliance.

Proper sample collection is essential to minimize preanalytical variability, which remains a significant contributor to inconsistent uACR results. In particular, the use of a first-morning void urine sample is strongly recommended, as it exhibits lower intra-individual variability and better reflects true 24 h albumin excretion compared to random daytime sampling [21–23]. At the same time, although uACR from a first-morning void is now the preferred method for albuminuria assessment in routine practice, 24 h urine collection may still be valuable in the nephrology setting. In particular, it provides important information on sodium and protein intake, which can influence the albuminuric response to renin–angiotensin system inhibition. Thus, in selected patients, especially those with suspected volume-dependent albuminuria, 24 h collections can support therapeutic decisions [24].

Because numerous factors influence albumin excretion, often leading to albumin overestimation in random samples compared with first-morning void samples, confirmation of elevated uACR value in random samples with a repeat test using a first-morning specimen is advised to reduce the risk of false positives [23, 25]. Recent findings highlight the clinical significance of low-level urine albumin excretion, e.g. uACR value > 10 mg/g significantly predicts CKD progression in type 2 diabetes, while a threshold of 16 mg/g in the general population is linked to higher cardiovascular mortality risk [26, 27]. Accordingly, the selection of appropriate analytical methods is critical. Quantitative immunoassays, based on immunoturbidimetric or immunonephelometric techniques, are preferred for their superior sensitivity in detecting low levels of albuminuria. In contrast, semiquantitative methods, such as dipstick analysis, are strongly discouraged due to their limited accuracy, especially in the low-to-moderate range of uACR [25, 28, 29]**.** An important challenge is the lack of uACR measurement standardization across different assays. Different calibration traceability can impair the transferability of uACR results across laboratories, thus compromising uACR clinical decision-making formerly based on globally recognized decisional values. Also, the lack of harmonization in reported units for uACR can impair result interpretation. To address this, international initiatives by the National Kidney Disease Education Program and the International Federation of Clinical Chemistry have been launched to support the standardization of albumin and creatinine measurement in urine with the recent release of a commutable Standard Reference Material (SRM 3666) to support In Vitro Diagnostics for effective traceability implementation [30]. In addition to technical considerations, the value of structured collaboration between laboratory professionals and clinicians should be emphasized. Laboratories can play a proactive role by providing guidance on appropriate test requisition (e.g., requesting uACR directly rather than separately ordering albumin and creatinine), educating clinicians on preanalytical pitfalls, and promoting consistent reporting practices—including the use of mg/g (or mg/mmol) as standard units, and the elimination of obsolete terminology such as “microalbuminuria” and “macroalbuminuria” [11, 31]. Collectively, these strategies can contribute to more reliable uACR assessment, improved diagnostic accuracy, and ultimately better risk stratification and management of CKD patients**.**

## Conclusion

The ALLIANCE project highlighted critical gaps in the current approach to CKD management, particularly in the use of uACR testing and referral practices. Addressing these challenges through enhanced clinician education, improved screening protocols, and early nephrology referral could substantially improve CKD outcomes and reduce the cardiovascular burden associated with albuminuria. Future efforts should prioritize the implementation of these recommendations to ensure more consistent and effective care for high-risk populations.

## Data Availability

All data generated or analyzed during this study are included in this published article. Further inquiries can be directed to the corresponding author.
